# When the Attack Comes Before the Asthma: Violence Increases Risk from Pollution

**DOI:** 10.1289/ehp.115-a416a

**Published:** 2007-08

**Authors:** Tina Adler

Chronic physical or emotional stress is known to impair the immune system, a link that may explain some of the health disparities found among different socioeconomic groups. Researchers have also found that living in poor, urban communities or near highways is linked to a greater risk of developing childhood asthma and other breathing problems. A new study now reveals that psychosocial stressors may increase children’s vulnerability to the effects of traffic-related air pollution **[*EHP* 115:1140–1146; Clougherty et al.]**.

The researchers examined data from the Maternal–Infant Smoking Study of East Boston, which began in 1987 to establish a cohort of pregnant women. In 1997, parents or guardians of 417 children of the cohort, then aged 4 to 11.5, answered questions about the children’s exposure to violence. Respondents were asked about the frequency with which the children had ever seen hitting, a shooting, or a stabbing, or heard domestic verbal abuse or gunshots. Other studies have suggested that residual trauma from witnessing episodic violence is a source of chronic stress for urban residents.

About 45% of the children had witnessed at least one violent act, and almost 20% had witnessed at least two. Responses were generalized to account for variables that can affect the severity of such acts, including whether the child knew the victim or perpetrator.

The researchers then acquired data collected between 1987 and 2004 on Boston levels of nitrogen dioxide (NO_2_), a constituent of vehicular exhaust with a known link to asthma. They used computerized mapping tools to estimate NO_2_ exposure at the children’s residences in East Boston, a working-class urban neighborhood with highways running through it.

About 25% of the children in the study had asthma. However, residential exposure to NO_2_ was linked to asthma only among children who were above the median for exposure to violence. The association between asthma and NO_2_ exposure disappeared when the researchers looked at the group as a whole.

The authors conclude that their findings “indicate ancillary effects of violence on children in addition to direct injury and post-traumatic stress.” Larger studies are needed to investigate other possible interactions among risk factors for asthma. It is also important to study the effects of other pollutants, including indoor air pollution. The authors observe that accurate reports about violence are difficult to obtain, and that violence exposures may be a sign of other problems with family stability that affect stress levels and health.

## Figures and Tables

**Figure:**
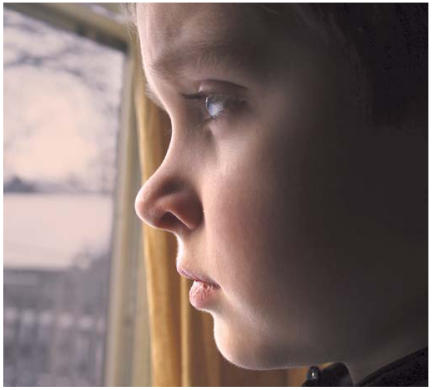
Added burden Stress can compound the physiological effects of environmental pollutants such as nitrogen dioxide.

